# Efficacy of Local N-Acetylcysteine Administration in Mitigating OHSS Parameters: A Comparative Analysis With Dopaminergic Agonist in the OHSS Model

**DOI:** 10.1155/ije/1634072

**Published:** 2024-12-05

**Authors:** Dulce Elena Letras-Luna, Nora Hilda Rosas-Murrieta, Nidia Gary Pazos-Salazar, Jorge Flores-Hernández, Francisco Castelán, Berenice Venegas, Alfonso Díaz, Samuel Treviño, Daniel Juárez-Serrano, Wendy Argelia García-Suastegui, Anabella Handal-Silva, José Luis Morán-Perales

**Affiliations:** ^1^Department of Biology and Reproductive Toxicology, Institute of Sciences (ICUAP), Benemérita Universidad Autónoma de Puebla, Puebla, Mexico; ^2^Biochemistry and Molecular Biology Laboratory, Chemistry Center, Institute of Sciences (ICUAP), Benemérita Universidad Auténoma de Puebla, Puebla, Mexico; ^3^Department of Microbiology, Faculty of Chemical Sciences, Benemérita Universidad Autónoma de Puebla, Puebla, Mexico; ^4^Institute of Physiology, Benemérita Universidad Autónoma de Puebla, Puebla, Mexico; ^5^Department of Cell Biology and Physiology, Institute of Biomedical Research, Universidad Nacional Autónoma de México, Mexico City, Mexico; ^6^Tlaxcala Center for Behavioral Biology, Universidad Autónoma de Tlaxcala, Tlaxcala, Mexico; ^7^Faculty of Biological Science, Benemérita Universidad Autónoma de Puebla, Puebla, Mexico; ^8^Laboratory of Metabolomics and Chronic Degenerative Diseases, Institute of Physiology, Benemérita Universidad Autónoma de Puebla, Puebla, Mexico; ^9^Department of Biochemistry-Food Science, Faculty of Chemical Sciences, Benemérita Universidad Autónoma de Puebla, Puebla, Mexico

**Keywords:** antioxidant, cabergoline, dopamine agonist, intrabursal injection, N-acetylcysteine, ovarian hyperstimulation, oxidative stress, VEGF

## Abstract

In this study, we evaluated the effects of intrabursal administration of cabergoline and N-acetylcysteine on ovarian hyperstimulation syndrome (OHSS) in an immature rat model. The study assessed body, ovarian, and uterine weights, as well as the concentrations of vascular endothelial growth factor A (VEGF-A). Moreover, levels of MDA, 4-HDA, and nitrites were assessed in ovarian homogenates, and vascular permeability was quantified in the peritoneal cavity. Ovarian morphology was characterized using histology and hematoxylin–eosin staining, determining the count of ovarian follicles and corpus luteum. Our results demonstrated a significant increase in lipoperoxidation, nitrite levels, and VEGF-A concentrations in the OHSS group compared to the control group. These biochemical alterations corroborate the successful induction of OHSS in the experimental model. Direct injection into the ovarian bursa resulted in reduced vascular permeability and VEGF-A levels, suggesting that the effects of cabergoline are predominantly ovarian. Particularly, cabergoline did not significantly alter other parameters such as ovarian weight, lipoperoxidation, nitrite levels, or morphology. Conversely, low concentrations of N-acetylcysteine (25–50 µg/kg) significantly reduced ovarian and uterine weights, VEGF-A levels, and vascular permeability. Interestingly, this dose–response relationship was not observed at higher NAC concentrations (100–200 μg/kg), suggesting a potential threshold beyond which NAC loses efficacy in these specific parameters. Our results suggest that the localized administration of N-acetylcysteine shows promise as a therapeutic strategy for OHSS by modulating key parameters associated with the syndrome. These promising results warrant further investigation into its mechanisms and efficacy, potentially expanding therapeutic options for OHSS management.

## 1. Introduction

Ovarian hyperstimulation syndrome (OHSS) represents an iatrogenic condition precipitated by assisted reproductive technology. It is marked by a spectrum of clinical manifestations, including hemoconcentration, ascitic fluid accumulation, and disturbances in electrolyte balance. The severity of OHSS can vary considerably, extending from mild abdominal bloating and discomfort to critical complications such as renal impairment, and in extreme cases, it can lead to life-threatening outcomes such as thromboembolism or multisystem organ failure [1].

Vascular endothelial growth factor type A (VEGF-A) plays a crucial role in the pathogenesis of OHSS. Studies using a murine model have shown that the administration of gonadotropins in varying doses increases VEGF-A secretion and VEGF receptor 2 (VEGFR-2) expression, leading to increased vascular permeability [2]. These effects were not replicated in ovariectomized animals receiving gonadotropins, which underscores the ovary as the main site of gonadotropin-induced hyperstimulation [3]. In this murine model, cabergoline, a dopamine receptor 2 (DAR2) agonist, reduced vascular permeability at low doses without impeding the proliferation of luteal blood vessels [4]. Furthermore, DAR2 agonism curtailed vascular permeability by diminishing ovarian VEGF-A secretion. However, cabergoline did not affect ovarian VEGF-A mRNA expression levels in OHSS rats [5].

In the clinical setting, cabergoline has been shown to decrease the volume of ascites and vascular permeability [6]. While DAR2 agonism has been associated with a reduced incidence of OHSS, it did not significantly decrease the occurrence of severe OHSS cases [7]. A prospective randomized study indicated that cabergoline administration in women undergoing assisted reproductive technology was associated with a reduced occurrence of early OHSS [8]. Low-dose dopamine agonists have been reported to decrease the risk of developing OHSS by nearly 50% [9]. However, while cabergoline has been effective in reducing early OHSS in gonadotropin release hormone (GnRH) agonists *in vitro* fertilization (IVF) cycles, it has not been able to prevent severe cases of OHSS [10].

Reactive oxygen species (ROS) are implicated in over one hundred pathological conditions. Regarding reproductive health, elevated levels of ROS are observed in disorders such as polycystic ovary syndrome (PCOS), endometriosis, and infertility [11]. VEGF-A has also been shown to increase ROS production in human umbilical vein endothelial cells (HUVECs). In a related study, cells with silenced NOX2 or NOX4 exhibited reduced ROS production, cellular proliferation, and migration. Intriguingly, in endothelial cells, NOX4-derived hydrogen peroxide (H_2_O_2_) can activate NOX2, which then promotes mitochondrial ROS production through pSer36-p66Shc. This cascade further amplifies ROS-dependent VEGFR-2 signaling [12].

Furthermore, dopamine metabolism is related to increased ROS production and oxidative stress, which are attributed to the increased activity of nicotinamide adenine dinucleotide phosphate (NADPH) oxidase [13, 14], monoamine oxidase (MAO), and dopamine transporter (DAT) [15]. However, DAR2 agonists are recognized for their antioxidant properties. Specifically, cabergoline administration has been found to increase glutathione (GSH) system activity in the striatum, providing neuroprotective effects [16]. Furthermore, DAR2 knockout mice (DAR2−/−) show increased oxidative stress [13].

Furthermore, a study investigating patients undergoing intracytoplasmic sperm injection (ICSI) who developed severe symptoms of OHSS revealed a notable increase in serum malondialdehyde (MDA) levels, among other indicators of oxidative stress, compared to those of healthy women [17]. This rise suggests that oxidative stress may contribute to the pathogenesis of OHSS. In this context, antioxidants may offer a viable therapeutic alternative for OHSS. Accordingly, the objective of our study was to evaluate the effectiveness of localized administration of N-acetylcysteine (NAC) or cabergoline in mitigating ovarian adverse effects associated with OHSS in an animal model. Our results demonstrate that low-dose antioxidant treatment significantly reduces the key parameters of OHSS, surpassing the effects of cabergoline in certain aspects.

## 2. Materials and Methods

### 2.1. Hormonal Treatment Protocols

This study utilized 22-day-old immature female Wistar rats, which were divided into three experimental groups. Distinct protocols were used to induce and assess the effects of OHSS, treating each group separately. We describe the groups and their corresponding procedures as follows: (1) Intact group (*n* = 6): At 9:00 h, this group received an injection of 0.9% isotonic saline solution (SSI 10 UI) on the 24th and 26th days of life. (2) The rats in the control group (*n* = 6) received an injection of pregnant mare serum gonadotrophin (PMSG, Zoetis q-1196-421, Mexico) (10 UI) on the 24th day of life at 9:00 h, followed by an injection of human chorionic gonadotropin (hCG) (Sigma, C-0434, Germany) (10 UI) 48 h later on the 26th day of life at 9:00 h. (3) OHSS group (*n* = 6): The OHSS group received PMSG injections (10 UI) from the 22nd to the 25th day of life at 9:00 h daily. On the 26th day of life, an hCG injection (10 UI) was administered at 9:00 h (Table 1). All procedures and protocols used in this investigation adhered to the national (NOM-062-ZOO-1999) and international guidelines for the care and use of experimental animals.

### 2.2. Intrabursal Injection Procedure

Twenty-four hours after hCG administration, we administered the selected doses of NAC or cabergoline intrabursally to Wistar rats. The doses of NAC were 25, 50, 100, and 200 *μ*g/kg (micrograms per kilogram) of body weight. The doses of cabergoline were 20, 40, 60, and 100 µg/kg of body weight (Table 1). First, we weighed each rat to calculate the right amount of each substance. We then multiplied the weight by the required dose to determine the total amount of substance needed. Next, we prepared the solutions at the appropriate concentrations to ensure that injecting a volume of 40 *μ*L would deliver the correct dose. We administered the substances locally, dividing the total volume of 40 μL into two injections of 20 μL in each ovary. In groups of rats affected by OHSS, we performed laparotomies to expose their right and left ovaries. We used a motorized nanoinjector (Stoelting, Co., USA) with a 100 μL microsyringe (Hamilton, USA) and a 29-gauge needle. A 20 μL solution of saline (0.9% SSI) was injected bilaterally into the ovarian bursa at 4 *μ*L/min. Furthermore, we calculated and administered cabergoline (Sigma C0246, USA) or NAC (Sigma A7250, USA) doses based on the body weight of the rats. We held the needle in place for 2 min after the injection to prevent leakage. We then cleaned and dried the ovaries before placing them back into the abdominal cavity. We sutured the skin and muscle layers to close the incisions. We consistently conducted all surgical procedures between 9:00 and 10:00 h. After surgery, rats were humanely euthanized after 24 h for tissue analysis. We evaluated four distinct concentrations of both the dopaminergic agonist and the antioxidant to determine their effects on OHSS. A control group received a saline solution (0.9% SSI) for comparison, and a SHAM group underwent all surgical procedures except the actual treatment to serve as an additional control.

### 2.3. Measurement of Serum VEGF-A Concentration

The concentration of serum VEGF-A was measured using a sandwich enzyme-linked immunosorbent assay (ELISA) specifically developed for rat VEGF-A (Sigma, RAB0511, USA). Before the assay, serum samples were prepared according to the manufacturer's protocol. The ELISA kit is highly specific to rat VEGF-A, with antibodies not showing cross-reactivity with other soluble proteins or immunoreactive substances. This specificity ensures reliable and consistent results. The detection limit of the assay starts at 2 pg/mL, with a standard curve typically ranging from 2–200 pg/mL The results from the assay were recorded and are reported in picograms per milliliter (pg/mL) of serum. All measurements were performed in duplicate, as recommended by the guidelines.

### 2.4. Assessment of Ovarian and Uterine Mass

The ovarian and uterine masses were assessed in this study. We meticulously documented the body weight of each animal before euthanasia. The ovaries and uterus were carefully removed and weighed. The uterine mass was determined immediately after excision. The right and left ovarian tissues were separately weighed using a precision analytical balance and the measurements were recorded. For analytical purposes, the mean mass of the paired ovaries was calculated and used in subsequent evaluations.

### 2.5. Homogenization of Ovarian Tissue Samples

The ovarian homogenates underwent the following processing steps: The ovaries were homogenized from each experimental group for protein extraction by pulverizing them in liquid nitrogen with a mortar and pestle. Following resuspension in phosphate-buffered saline (PBS), we centrifuged the mixture at 4°C for 45 min at 12,000 g. We meticulously gathered the supernatant and disposed of the resultant pellet. We quantified the protein concentrations in the supernatant utilizing the Bradford protein assay (Bio-Rad, Hercules, California, USA), adhering to the manufacturer's instructions.

### 2.6. Nitrite Determination

Nitrite (NO^−2^) levels in the supernatant were quantified to assess the presence of nitric oxide (NO). Due to the volatile nature of NO, arising from its high reactivity and short half-life, we measured its stable metabolic product, nitrite, using the colorimetric Griess assay. The assay was initiated by mixing 100 *μ*L of tissue supernatant with 100 *μ*L of freshly prepared Griess reagent (comprising 0.1% N-(1-naphthyl)ethylenediamine dihydrochloride and 1.32% sulfanilamide at a 1:1 ratio in 60% acetic acid, at 4°C). The resulting absorbance of the chromophore was measured at 540 nm using a SmartSpec 3000 spectrophotometer (Bio-Rad, Hercules, California, USA). The NO^−2^ concentration in the samples was determined by interpolation from a standard NaNO_2_ curve prepared under identical conditions, with concentrations ranging from 1 to 10 *μ*M. To account for age-dependent variations in NO^−2^ levels, presumably reflecting the age of the organism from which the tissue was derived, or to ensure consistency, NO^−2^ and lipoperoxidation levels were normalized to protein concentrations. The protein concentrations were determined using the Bradford assay, and no significant differences were found between experimental groups.

### 2.7. Evaluation of Lipoperoxidation Levels

Levels of MDA and 4-hydroxyalkenals (MDA + 4-HDA) were quantified in 200 μL of the same supernatant used for the previous analysis. The colorimetric assay involved mixing 200 μL of this supernatant with 650 μL of 10.3 mM N-methyl-2-phenylindole, which was prepared in a mixture of acetonitrile:methanol (3:1), followed by the rapid addition of 150 μL of methanesulfonic acid using a glass pipette to acidify the mixture, thus promoting the formation of a color complex. The mixture was vortexed, incubated at 45°C for 1 h in a water bath, and then centrifuged at a relative centrifugal force equivalent to 3000 rpm for 10 min. The absorbance of the transparent supernatant was quantified at 586 nm utilizing a SmartSpec 3000 spectrophotometer (Bio-Rad, Hercules, California, USA). The concentrations of MDA and 4-HDA in the samples were determined using a standard curve of 1,1,3,3-tetramethoxypropane, with concentrations varying from 0.5 to 5 *μ*M. Changes resulting from fluctuations in ovarian weight were clarified by standardizing the values to the protein concentration per milligram of tissue.

### 2.8. Ovarian Histology and Follicle Counting

The ovaries were entirely sectioned to quantify the distribution of follicles and corpora lutea (CLs), with results presented as percentages. Ovary samples were immediately fixed in Bouin's solution to preserve tissue morphology, sequentially dehydrated, and embedded in paraffin. Sections of 10 *μ*m thickness were prepared and stained with hematoxylin and eosin (H&E) for structural visualization. The stained sections were analyzed using a light microscope with a digital camera at 10x magnification. Follicles were enumerated and classified into three categories according to their developmental stages: preantral follicles (PFs), antral follicles (AFs), and preovulatory (POs). CLs were enumerated in each section. Each section was systematically analyzed to quantify the ovarian structures, and the results were presented as the percentage of each type in relation to the total number of identified ovarian structures, with the total of all structures comprising 100%.

### 2.9. Statistical Analysis

The results are expressed as the mean ± standard error of the mean (SEM). Data analysis was performed using IBM SPSS Statistics (IBM Corp., Armonk, New York, USA). The Shapiro–Wilk and Levene's tests were used to determine the normality and equality of error variances, respectively. For normally distributed data, statistical significance was determined using a one-way analysis of variance (ANOVA). Post hoc analyses were performed using Tukey's test for groups with homogenous variances, and Games–Howell's test for groups with unequal variances, while Student's *t*-tests were used for comparing follicular distribution between the OHSS group and the control group. A *p* value of < 0.05 was considered to indicate statistical significance. Statistically significant differences between experimental groups were represented as follows: ⁣^∗^*p* < 0.05, ⁣^∗∗^*p* < 0.01, and ⁣^∗∗∗^*p* < 0.001.

## 3. Results

### 3.1. Validation of the OHSS Model

**Figure 1:**
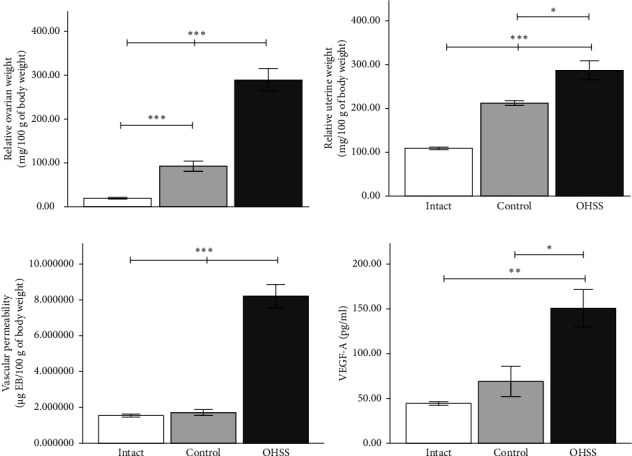
Physiological markers in OHSS rats. (a) Relative ovary weight. The OHSS group exhibited a significant increase in ovary weight compared to the intact and control groups. (b) Relative uterine weight. OHSS rats had significantly higher uterine weight compared to the intact and control groups. (c) Vascular permeability in OHSS animals was markedly elevated compared to the other groups. (d) Serum VEGF-A concentration in OHSS rats was significantly higher compared to the intact and control groups. Each bar represents the mean ± S.E.M. (*n* = 6). Statistical significance is indicated by ⁣^∗^*p* < 0.05, ⁣^∗∗^*p* < 0.01, and ⁣^∗∗∗^*p* < 0.001, as determined by one-way ANOVA.

In this study, an OHSS model was successfully established, as evidenced by significant increases in physiological markers such as ovarian weight, uterine weight, and abdominal vascular permeability. Furthermore, a marked increase in VEGF-A levels was observed (Figure 1), further confirming the validity of the model. Compared to the intact group which did not receive gonadotropin stimulation, the control group exhibited substantial increases in ovarian and uterine weight. However, these alterations were not accompanied by changes in VEGF-A concentration or vascular permeability, which affirms the role of the control group as a consistent baseline for comparison. The absence of changes in the intact group confirms the specificity of the OHSS induction method.

### 3.2. OHSS Alters Follicular Distribution and Increases Corpus Luteum Formation

**Figure 2:**
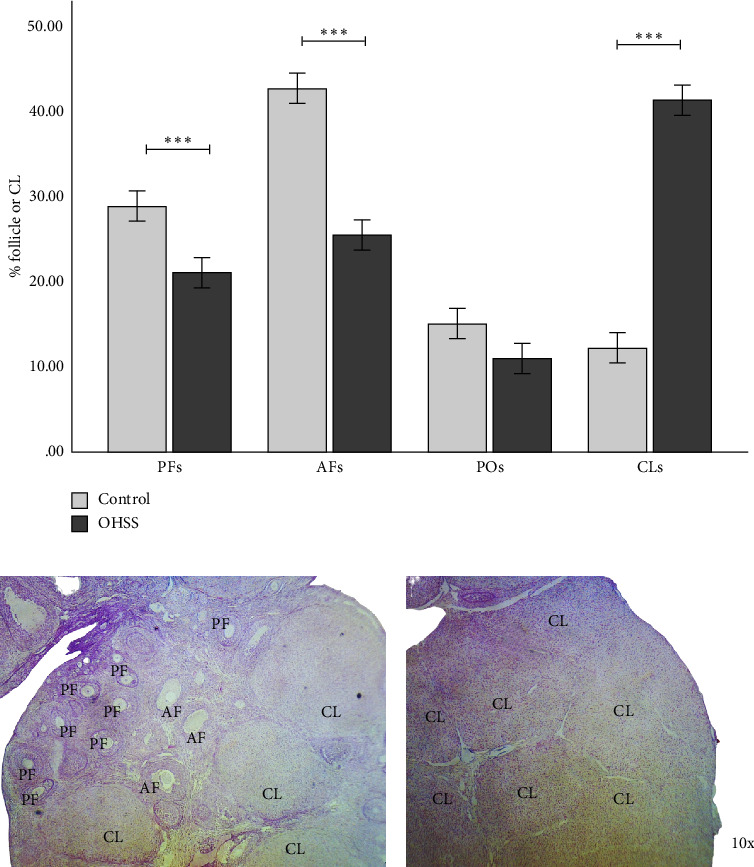
Follicular dynamics and corpus luteum distribution in control and OHSS-affected animals. (a) Percentage of follicles or CLs in the control and OHSS groups. The OHSS group showed a significant reduction in the percentage of preantral (PFs) and antral follicles (AFs), and a significant increase in the percentage of corpora lutea (CLs) compared to the control group. No significant changes were observed in preovulatory (POs) follicles. (b) Representative histological image of ovarian morphology from the control group. (c) Representative histological image of ovarian morphology from the OHSS group. Each bar represents the mean ± S.E.M. (*n* = 6). Statistical significance is indicated by ⁣^∗^*p* < 0.05, ⁣^∗∗^*p* < 0.01, and ⁣^∗∗∗^*p* < 0.001, as determined by Student's *t*-test.

The impact of OHSS on ovarian morphology was assessed in a rat model by utilizing the HE staining technique on ovarian tissue sections to enumerate follicles at various developmental stages. The analysis revealed significant alterations in the follicular dynamics of OHSS rats compared to the control group. In particular, the OHSS group demonstrated a substantial decrease in the percentage of PFs and AFs. Conversely, the OHSS group exhibited a substantially higher percentage of CL structures (Figure 2). The quantity of POs between the groups did not exhibit any significant differences. These findings underscore significant alterations in ovarian tissue structure caused by OHSS, suggesting a disruption in normal folliculogenesis. The ovary's function has been altered as evidenced by the accumulation of CLs and the reduction in PFs and AFs.

### 3.3. Elevated Markers of Oxidative Stress Attributable to OHSS

**Figure 3:**
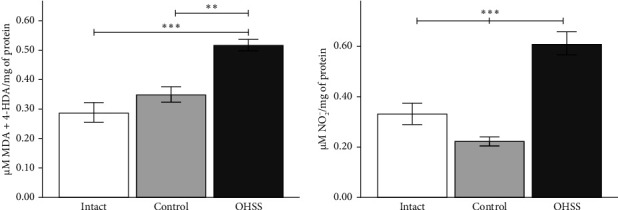
Oxidative stress markers in OHSS rats. (a) Lipoperoxidation levels in the OHSS rat model. The OHSS group exhibited significantly higher lipoperoxidation levels compared to the control and intact groups. (b) Nitrite concentration in the ovaries of OHSS rats was significantly elevated compared to the control and intact groups. No significant differences were observed between the control and intact groups for either marker. Each bar represents the mean ± S.E.M. (*n* = 6). Statistical significance is indicated by ⁣^∗^*p* < 0.05, ⁣^∗∗^*p* < 0.01, and ⁣^∗∗∗^*p* < 0.001, as determined by one-way ANOVA.

Rats with OHSS had considerable biochemical alterations, particularly in indicators of oxidative stress. The OHSS group had significantly greater levels of lipoperoxidation, as demonstrated by higher MDA + 4-HDA concentrations, than the intact and control groups (Figure 3(a)). The OHSS group showed significantly higher nitrite measures compared to the other groups (Figure 3(b)). These results suggest that oxidative stress is an important metabolic abnormality related to OHSS. Crucially, we found no significant variations in lipoperoxidation or nitrite levels between the control and intact groups, implying a direct relationship between oxidative stress and OHSS.

### 3.4. Cabergoline and NAC Reduce Vascular Permeability in a Dose-Dependent Manner Through Decreased VEGF-A

**Figure 4:**
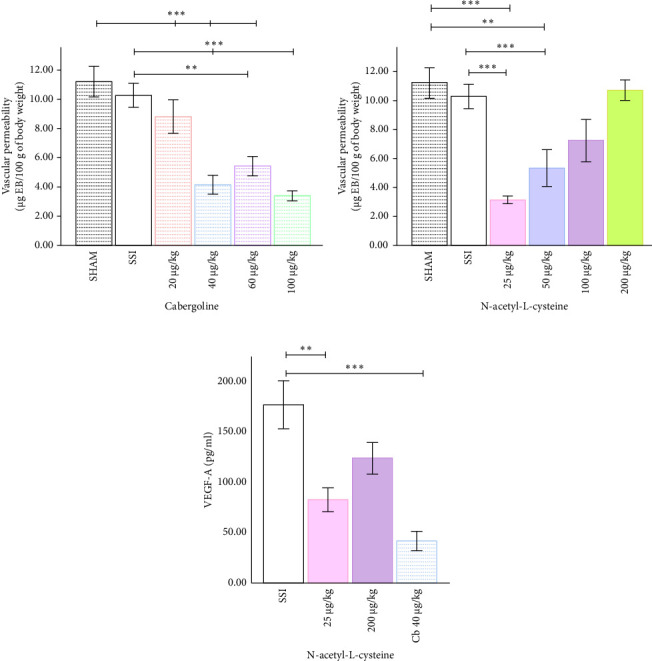
Differential impact of cabergoline and N-acetyl-L-cysteine (NAC) on OHSS-induced vascular permeability and serum VEGF-A levels in the OHSS rat model. (a) Vascular permeability in the ovary of rats treated with cabergoline. Cabergoline significantly reduced ovarian vascular permeability starting at a dose of 40 µg/kg, with the reduction maintained up to 100 µg/kg, compared to the saline solution (SSI) group. (b) Vascular permeability in rats treated with NAC. NAC effectively reduced vascular permeability at doses of 25 µg/kg and 50 µg/kg but had no significant effect at higher doses. (c) Serum VEGF-A concentration. NAC reduced VEGF-A levels significantly at 25 µg/kg, but no significant reduction was observed at 200 µg/kg. Cabergoline reduced VEGF-A levels at 40 µg/kg. Each bar represents the mean ± S.E.M. (*n* = 6). Statistical significance is indicated by ⁣^∗^*p* < 0.05, ⁣^∗∗^*p* < 0.01, and ⁣^∗∗∗^*p* < 0.001, as determined by one-way ANOVA.

Cabergoline administration significantly reduced ovarian vascular permeability in a dose-dependent manner. The vascular permeability was significantly lower in the 40 µg/kg group compared to the SSI and SHAM groups. The decreases were even greater at 60 µg/kg and 100 µg/kg (Figure 4(a)). Serum VEGF-A levels also decreased in tandem with this reduction in vascular permeability (Figure 4(c)). In the same way, NAC decreased vascular permeability starting at a dose of 25 µg/kg and stayed that way until 50 µg/kg, compared to the SSI and SHAM groups (Figure 4(b)). However, higher NAC doses of 100 µg/kg and 200 µg/kg did not result in significant further reductions in vascular permeability. VEGF-A levels were lowered significantly by NAC at a dose of 25 µg/kg, but there were no significant changes seen at the highest dose of 200 µg/kg (Figure 4(c)). Cabergoline, on the other hand, also led to a significant reduction in VEGF-A levels at 40 µg/kg. The OHSS, SSI, and SHAM groups did not significantly differ in vascular permeability or VEGF-A levels.

### 3.5. High Doses of NAC Mitigate Lipoperoxidation and Nitrite Levels, Exhibiting Contrasting Effects Compared to Cabergoline

**Figure 5:**
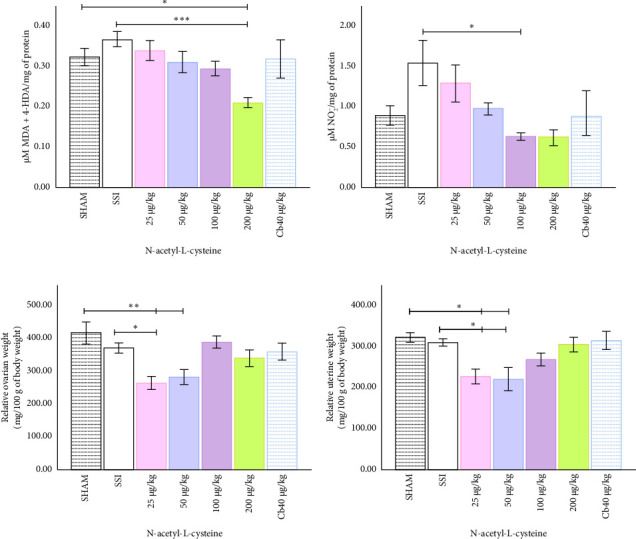
Effects of local N-acetyl-L-cysteine (NAC) and cabergoline administration on relative organ weights, lipoperoxidation, and nitrite levels in OHSS rats. (a) Lipoperoxidation levels (MDA + 4-HDA). A marked reduction in lipoperoxidation was observed at the 200 µg/kg dose compared to the SSI and SHAM groups. (b) Nitrite levels were reduced at the 100 µg/kg concentration compared to the SSI group. (c) Relative ovarian weight. NAC administration significantly reduced ovarian weight at the 25 µg/kg and 50 µg/kg doses compared to the saline solution (SSI) group. (d) Relative uterine weight. NAC significantly decreased uterine weight at the 25 µg/kg dose compared to the SSI group, with no significant changes at higher doses. Each bar represents the mean ± S.E.M. (*n* = 6). Statistical significance is indicated by ⁣^∗^*p* < 0.05, ⁣^∗∗^*p* < 0.01, and ⁣^∗∗∗^*p* < 0.001, as determined by one-way ANOVA.

NAC administration at a dose of 200 µg/kg significantly reduced lipoperoxidation levels (MDA + 4-HDA) in OHSS rats compared to both the SSI and SHAM groups (Figure 5(a)). This suggests that NAC has a strong antioxidative effect at high doses. In contrast, lipoperoxidation or nitrite levels did not change significantly at lower doses of NAC (25 µg/kg and 50 µg/kg) (Figures 5(a) and 5(b)). This suggests that the antioxidative effects are stronger at higher concentrations. However, at all tested doses, cabergoline did not significantly alter the levels of lipoperoxidation or nitrite (Figures 5(a) and 5(b)).

### 3.6. Compared to the Dopaminergic Agonist, NAC Attenuates OHSS-Induced Alterations in Ovarian and Uterine Parameters With Contrasting Effects

In addition to its antioxidative effects, NAC significantly reduced ovarian weight at doses of 25 µg/kg and 50 µg/kg relative to body weight compared to the SSI and SHAM groups (Figure 5(c)). The 25 µg/kg dose significantly reduced uterine weight compared to the SSI group (Figure 5(d)). However, higher doses of NAC did not result in further reductions in ovarian or uterine weights, indicating that lower doses were more effective in mitigating these structural changes. In contrast, cabergoline had no significant effect on ovarian or uterine weights at any of the tested doses, emphasizing the distinct effects of NAC and the dopaminergic agonist.

### 3.7. NAC Attenuates OHSS-Induced Alterations in Ovarian Morphology and Offers Contrasting Effects to Dopaminergic Agonist

**Figure 6:**
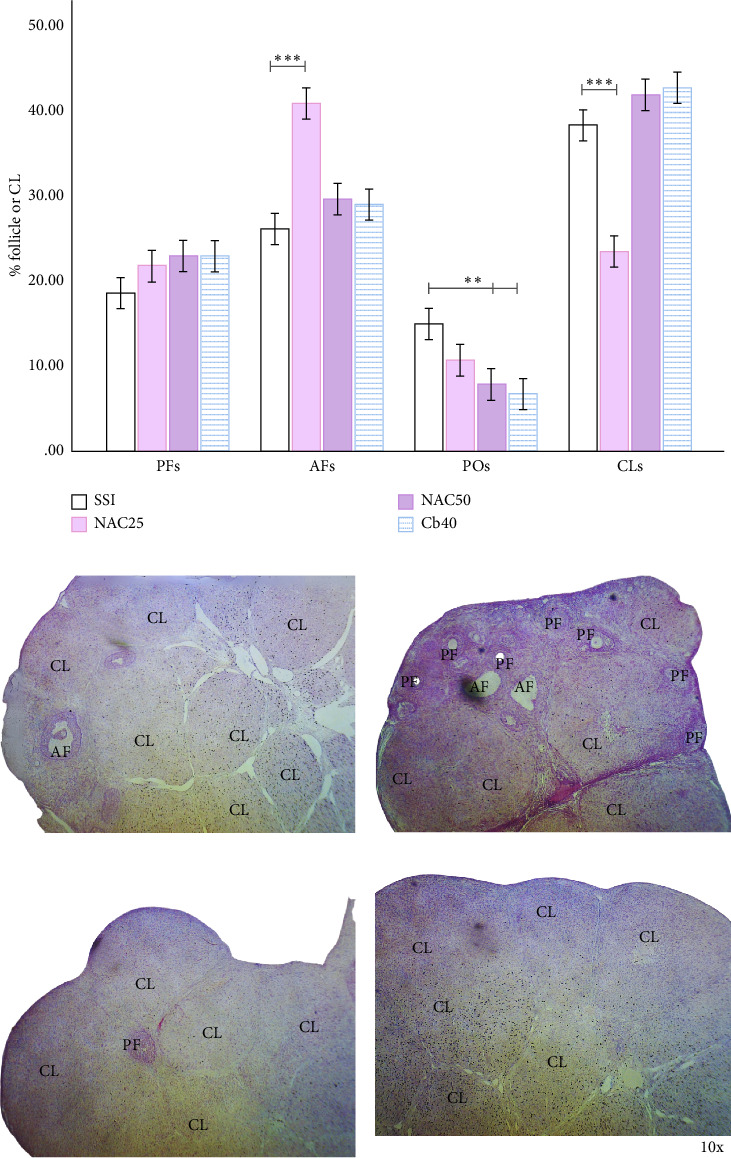
Effects of local N-acetyl-L-cysteine (NAC) and cabergoline (CB) administration on follicular dynamics and corpora lutea (CLs) distribution in OHSS rats. (a) Percentage of follicles and CLs in the saline solution (SSI) group and NAC 25 µg/kg, NAC 200 µg/kg, and cabergoline 40 µg/kg (CB40) groups. The NAC 25 µg/kg group showed a significant increase in antral follicles (AFs) compared to the SSI group. Preovulatory (POs) follicles were significantly reduced in the CB40 and NAC 200 µg/kg groups, while corpora lutea (CLs) were reduced in the NAC 25 µg/kg group. (b) Representative histological image from the SSI group. (c) Representative histological image from the NAC 25 µg/kg group. (d) Representative histological image from the NAC 200 µg/kg group. (e) Representative histological image from the cabergoline 40 µg/kg group. Each bar represents the mean ± S.E.M. (*n* = 6). Statistical significance is indicated by ⁣^∗^*p* < 0.05, ⁣^∗∗^*p* < 0.01, and ⁣^∗∗∗^*p* < 0.001, as determined by one-way ANOVA.

NAC administration at 25 µg/kg resulted in a significant increase in AFs and a decrease in CLs compared to the SSI group (Figure 6(a)). However, at the higher dose of 200 µg/kg, NAC reduced POs without affecting AFs or CLs. Cabergoline (40 µg/kg) significantly reduced POs and CLs, comparable to NAC 200 µg/kg (Figure 6(a)). Histological images from the SSI, NAC 25 µg/kg, NAC 200 µg/kg, and cabergoline 40 µg/kg groups demonstrate the morphological changes (Figures 6(b), 6(c), 6(d), and 6(e)).

## 4. Discussion

In our study, we successfully established a rat model of OHSS that replicated the conditions defined by established methodologies [5]. This model is characterized by elevated levels of VEGF-A and increased vascular permeability, key features observed in OHSS. These changes are further accompanied by significant increases in body, uterine, and ovarian weights [18], which validate the model's accuracy. The control group received gonadotropins at lower doses, which is consistent with the doses in comparative studies [19]. Upon examination, the control group showed an increase in ovarian and uterine weights compared to the intact group [2, 5]. Importantly, the control group did not show increases in vascular permeability, VEGF-A levels, nitrite levels, or lipid peroxidation, confirming that the experimental group specifically induced OHSS characteristics.

In our OHSS-induced model, we observed a significant elevation in nitrite levels, which directly reflects increased NO production. This elevation is significant because NO is a potent vasodilator and increases vascular permeability. The observed increase in vascular permeability in OHSS is closely related to the upregulation of VEGF-A and activation of its receptor, VEGFR-2 [5]. Specifically, phosphorylation of tyrosine residues 949 (Y949) and 951 (Y951) on VEGFR-2 leads to eNOS activation, resulting in increased NO production. This activation triggers a cascade of signaling events in smooth muscle cells adjacent to the endothelium, critical for the phosphoinositide 3-kinase/protein kinase B (PI3K-Akt) signaling pathways, which depend on the vascular flow [20, 21]. These pathways are essential for vasodilation and increased vascular permeability [22] and play a significant role in the process of vascular remodeling [23]. Estrogens further influence these pathways by upregulating eNOS activity through Ser/Thr kinase activation [24, 25]. Elevated estrogen levels are a consistent finding in clinical cases of OHSS and in the corresponding animal models [18, 19, 26], which may elucidate the surge in the concentration of nitrite observed in our OHSS model.

The process of lipoperoxidation involves adding oxygen molecules to the unsaturated fatty acyl chains of lipids, which increases their hydrophilicity and diffusion to the membrane surface [27]. This alteration enables enzymes such as cyclooxygenases and lipoxygenases to access their lipid substrates, catalyzing the production of metabolites that drive inflammation and support the binding of proteins and receptors to oxidized lipids [28]. Aldehydes such as 4-hydroxynonenal (4-HNE) and MDA are made when lipid peroxides break down. They are important parts of this class [29]. These aldehydes have a big effect on the structure of the lipid bilayers because they change how the lipids interact with each other, the ion gradients, the fluidity, and the permeability [30, 31]. Furthermore, studies have shown that MDA concentrations ranging from 150 to 250 *μ*M can induce a dose-dependent reduction in the total electrical impedance of endothelial cell monolayers, which means that endothelial permeability increases [32].

OHSS upregulates the expression and activity of uncoupled NOS, causing a surge in NO and subsequent production of peroxynitrite (ONOO-). This event is a precursor to the formation of reactive nitrogen species (RNS) and inflammatory mediators, exacerbating oxidative stress [28]. Researchers found that women who had ICSI and then developed severe OHSS symptoms had significantly higher levels of MDA and other signs of oxidative stress than healthy women [17]. This clinical evidence underscores the role of oxidative stress in OHSS pathogenesis. The observed increase in nitrites and lipoperoxidation in our OHSS rat group reinforces this association, highlighting the relevance of targeting oxidative stress in OHSS management.

Antioxidants are essential for mitigating lipid peroxidation. They can terminate radical chain reactions, which are not enzyme-dependent, by interacting with alkyl-peroxyl or alkoxyl radicals [27]. Inspired by these principles, our research examined the effects of an antioxidant, specifically NAC, on OHSS. We observed that NAC reduced nitrite levels and lipid peroxidation at the highest dose administered. Furthermore, NAC provides a defense against ROS, slightly increasing H_2_S and sulfur species within the cell. This increase is due to the gradual release of cysteine from NAC, which promotes the continuous generation of sulfur products that confer protection beyond GSH [33]. However, the decrease in lipoperoxidation at these doses did not correspond with the decrease in vascular permeability.

Several factors, including the activation of eNOS and SRC proteins, influence vascular permeability, a hallmark of OHSS [22]. Within focal adhesions, signaling is mediated by transducers such as focal adhesion kinase (FAK) and its substrate paxillin. Although SRC activation can follow FAK, the SRC-mediated phosphorylation of FAK is essential for modulating cell adhesion and migration in response to VEGF-A [34]. SRC also modifies endothelial adherent junctions by phosphorylating VE-cadherin in response to VEGF-A [35]. This phosphorylation triggers VE-cadherin endocytosis, disrupting adherent junctions, and thus augmenting vascular permeability [36].

In a murine model, the agonism of DAR2 reduces vascular permeability by reducing VEGF-A secretion [5, 36, 37]. Cabergoline significantly reduces VEGFR-2 expression in endometrial cells in rats induced with OHSS [38]. In clinical settings, cabergoline administration has been associated with a reduction in ascites volume and vascular permeability [6]. Despite its ability to moderate the incidence of OHSS, the DAR2 agonist did not significantly alter the rates of severe OHSS [7]. Compared to other treatments such as letrozole, cabergoline was just as good at lowering the size of the ovaries, the amount of blood flow through them, and the amount of VEGF in their tissues, which shows that it helps lessen the effects of OHSS. However, it did not significantly alter VEGFR-2 expression more than other treatments [39]. Our data suggest that direct cabergoline administration to the ovarian bursa specifically decreases vascular permeability and VEGF-A levels, indicating a localized effect within the ovary. However, cabergoline did not affect other studied parameters, such as ovary weight, lipoperoxidation, nitrite ion levels, and ovarian morphology. In contrast, NAC administration at low concentrations (25–50 µg/kg) not only significantly reduced VEGF-A levels and vascular permeability but also decreased ovarian and uterine weights and favorably altered ovarian morphology by increasing AF counts and decreasing CL numbers. These findings suggest that NAC, at low doses, has a more comprehensive modulatory effect on key OHSS parameters than cabergoline, potentially offering superior therapeutic benefits.

NAC has also been shown to upregulate the expression of cell adhesion proteins, including VE-cadherin, occludin, and *β*-catenin, to protect against alterations induced by ionizing radiation [40]. Furthermore, VEGF-A typically stimulates the phosphorylation of VE-cadherin and -catenin, a process that NAC inhibits [36, 41]. Research on mice using ovarian autografts has revealed that NAC suppresses VEGF-A mRNA soon after grafting, leading to a significant rise 7 days later, which subsequently decreases when estrogens are present [42]. Low-dose antioxidant treatment in our study led to a reduction in VEGF-A concentration, which validates these findings. Interestingly, higher doses of NAC did not produce a reduction in VEGF-A levels or vascular permeability. This phenomenon may be due to a biphasic dose–response relationship, where low doses of NAC modulate signaling pathways that suppress VEGF-A expression and vascular permeability, while higher doses primarily exert antioxidative effects without further influencing these pathways. It suggests that NAC's efficacy in reducing vascular permeability and VEGF-A levels is optimal at lower doses, possibly due to its impact on redox-sensitive transcription factors that regulate VEGF-A expression. Moreover, that hypothesis is still to be studied. Therefore, careful consideration of dosing strategies is crucial to maximize NAC's therapeutic benefits in OHSS.

In this study, the OHSS group exhibited a notable increase in the number of CLs, which suggests a gonadotropin-induced elevation in ovulation rates compared to the control group. We also observed a decrease in the proportion of PFS and AFS, whereas the number of POs follicles was not significantly affected. These findings are in accordance with previous reports that have documented a rise in CLs and a reduction in follicular counts after OHSS induction in animal models [18, 43]. Correspondingly, OHSS in humans is characterized by the development of multiple CLs [44].

The intervention with NAC at a dose of 25 µg/kg markedly diminished ovarian and uterine weights. This outcome is associated with changes in ovarian morphology distribution, including a significant increase in PAs and AFs and a decrease in CLs, suggesting that NAC may positively influence the early stages of folliculogenesis and restore normal ovulatory function. In contrast, higher doses of NAC (200 µg/kg) reduced POs follicles without affecting AFs or CLs, indicating that NAC may have dose-dependent effects on follicular dynamics. Supporting our results, other research has indicated that NAC increases the number of PFs in ovarian autografts, which is consistent with the modulatory effects we observed on follicular development [42].

Regarding the use of antioxidants, although specific evidence on their use in preventing or treating OHSS is limited, research highlights the benefits of antioxidants such as NAC in ovarian stimulation contexts [45]. NAC has also been shown to upregulate the expression of cell adhesion proteins, including VE-cadherin, occludin, and β-catenin, thereby enhancing endothelial barrier function [40]. Recent studies have demonstrated that NAC protects endothelial cells from hyperpermeability induced by inflammatory cytokines by preserving tight junction integrity [46]. In addition, NAC mitigates oxidative stress-induced endothelial dysfunction, which is a key factor in the increased vascular permeability observed in OHSS [45].

Our findings suggest that localized administration of low-dose NAC may be effective in mitigating key parameters associated with OHSS, including reducing vascular permeability, VEGF-A levels, and ovarian and uterine weights. In addition, NAC showed beneficial effects on follicular dynamics. It is important to note that while higher doses of NAC did not affect VEGF-A levels or vascular permeability, they did reduce lipid peroxidation and nitrite levels, suggesting that NAC can provide significant benefits in managing oxidative stress even at higher concentrations.

While there are promising treatments for OHSS, none are entirely effective. Modulators of GnRH, such as GnRHa and GnRHanta, have shown utility but come with limitations. GnRH agonists are used to induce final oocyte maturation instead of HCG, significantly reducing the risk of OHSS by inducing rapid and reversible luteolysis [47]. On the other hand, GnRH antagonists help prevent OHSS by reducing vascular permeability and the expression of VEGF and its receptors in hyperstimulated ovaries. Studies have shown that GnRH antagonists are more effective than GnRH agonists in reducing estradiol levels and the expression of VEGF receptors [48]. In addition, the combination of GnRH agonists with the cryopreservation of all embryos (“freeze-all”) is a recommended strategy to prevent OHSS in high-risk patients, although cases of severe OHSS have been reported even with this technique [49].

Another promising treatment is letrozole. The combination of letrozole with prednisone has been shown to significantly reduce the incidence of severe OHSS in women treated with GnRH agonists for assisted fertilization, although the ongoing pregnancy rate was lower compared to the group treated with letrozole alone [50]. The administration of letrozole at doses of 7.5 mg reduced the incidence of moderate and severe OHSS in high-risk patients by dose-dependently lowering estradiol and VEGF levels [51]. However, a case of severe OHSS was reported in a woman with breast cancer and PCOS treated with letrozole and GnRH agonist, suggesting that these treatments do not eliminate the risk of OHSS [52].

A meta-analysis concluded that letrozole reduces the incidence of moderate and severe OHSS in high-risk women, although it did not show significant benefits in preventing mild OHSS [53]. Comparing letrozole with GnRH antagonists, a clinical trial found that both have similar effectiveness in preventing OHSS, with letrozole being more cost-effective and providing greater patient satisfaction [54]. In addition, a study in rats showed that letrozole and cabergoline are equally effective in preventing OHSS by reducing vascular permeability and VEGF levels [39].

In summary, while there are promising treatments for OHSS, such as GnRH modulators and letrozole, none are completely effective and have significant limitations [47, 54]. For example, GnRH agonists can reduce OHSS risk but are associated with lower pregnancy rates [49]. Letrozole has been shown to reduce OHSS incidence, but severe cases can still occur [52]. In this context, NAC emerges as a viable and promising therapeutic option due to its ability to reduce oxidative stress, improve the integrity of cellular junctions, and, at low doses, decrease vascular permeability and VEGF-A levels. These combined effects could mitigate factors contributing to OHSS development, potentially offering a valuable therapeutic intervention. Moreover, NAC has demonstrated benefits in improving oocyte quality and minimizing oxidative damage in animal models of ovarian hyperstimulation [45], suggesting additional potential in enhancing reproductive outcomes. These findings suggest that NAC may mitigate OHSS symptoms and enhance overall reproductive success, potentially making it a valuable adjunct therapy in fertility treatments.

While our findings are encouraging, certain limitations should be acknowledged. This study was conducted using an animal model, and thus, the results may not fully extrapolate to humans. In addition, the long-term effects of NAC administration were not assessed. Future studies should include clinical trials to confirm the efficacy and safety of NAC in human subjects and investigate the long-term outcomes of its use in OHSS management.

## 5. Conclusion

Our findings indicate that low-dose NAC administration may positively influence critical parameters of OHSS. Specifically, NAC reduced VEGF-A levels and vascular permeability, decreased ovarian and uterine masses, and altered ovarian morphology by promoting folliculogenesis and suppressing luteinization. These results suggest that NAC may serve as a beneficial therapeutic agent in the management of OHSS. Due to its favorable safety profile and cost-effectiveness, NAC may provide a viable and accessible alternative for patients susceptible to developing OHSS.

To scale these preclinical findings into clinical practice, it is imperative to undertake rigorously designed clinical studies that assess the efficacy and safety of NAC in humans. Such studies will ascertain optimal dosing strategies and evaluate the potential advantages of NAC in enhancing reproductive outcomes for patients undergoing fertility treatments. Incorporating NAC into standard OHSS prevention and treatment protocols may enhance patient care by offering an effective strategy to reduce OHSS risk while promoting successful reproductive outcomes.

## Figures and Tables

**Table 1:** Description of experimental groups and administered treatments.

Groups	Day 22 to 25 of age	Day 26 of age	Day 27 of age
Intact	10 IU SSI (day 24)	10 IU SSI	—
Control	10 IU PMSG (day 24)	10 IU hCG	—
OHSS	10 IU PMSG (daily)	30 IU hCG	—
CB20	10 IU PMSG (daily)	30 IU hCG	Cabergoline 20 µg/kg
CB40	10 IU PMSG (daily)	30 IU hCG	Cabergoline 40 µg/kg
CB60	10 IU PMSG (daily)	30 IU hCG	Cabergoline 60 µg/kg
CB100	10 IU PMSG (daily)	30 IU hCG	Cabergoline 100 µg/kg
NAC25	10 IU PMSG (daily)	30 IU hCG	N-acetylcysteine 25 µg/kg
NAC50	10 IU PMSG (daily)	30 IU hCG	N-acetylcysteine 50 µg/kg
NAC100	10 IU PMSG (daily)	30 IU hCG	N-acetylcysteine 100 µg/kg
NAC200	10 IU PMSG (daily)	30 IU hCG	N-acetylcysteine 200 µg/kg
SSI	10 IU PMSG (daily)	30 IU hCG	Isotonic saline solution
SHAM	10 IU PMSG (daily)	30 IU hCG	SHAM surgery
	Subcutaneous injection	Subcutaneous injection	Microinjection

*Note:* Pregnant mare serum gonadotropin (PMSG) and human chorionic gonadotropin (hCG) were used for hormonal treatments, while cabergoline (CB) and N-acetylcysteine (NAC) were administered on day 28 by microinjection into the ovarian bursa.

## Data Availability

The data used to support the findings of this study are included within the article. Further inquiries can be directed to the corresponding authors.
